# Transforming Supply Logistics for Health Commodity Security in Africa

**DOI:** 10.9745/GHSP-D-23-00218

**Published:** 2024-02-28

**Authors:** Ebenezer Kwabena Tetteh

**Affiliations:** a Department of Pharmacy Practice and Clinical Pharmacy, School of Pharmacy, College of Health Sciences, University of Ghana, Accra, Ghana.

## Abstract

Current efforts to ensure health commodity security in Africa must be extended to include transforming existing public and private logistics infrastructure for inventory management into a state of prudent multiplicity while also considering efforts to improve product selection, demand quantification, procurement, and service delivery.

## Key Messages

- To resolve problems of ensuring secure supplies of all health commodities, health planners in African countries must first identify arrangements that best serve the public interests of promoting choice and competition in ensuring health commodity security.
- Investments in inventory management should not be viewed as a one-off exercise but rather as a continuous search for the optimal scale and scope of operations that ensure the availability of essential health commodities most of the time.
- Without competing alternatives to manage inventory, public-sector logistics monopolies lack adequate incentives to devise ways of reducing costs and improving output. Further, these monopolies make it more difficult to minimize the impact and duration of catastrophic supply disruptions.
- Current efforts to improve public-sector supply logistics must be extended to include the transformation of existing public and private logistics infrastructure for inventory management into a state of prudent multiplicity—one in which there are at least 2 full-line national logistics institutions competing to serve all government, nongovernmental, and private health facilities.
- Health planners should consider creating a state of prudent multiplicity in their roadmaps, master plans, and health system strengthening initiatives.

## INTRODUCTION

To guide their efforts to build population health by strengthening public-sector logistics systems (or supply chains), health planners in Africa can use the evolutionary path or roadmap suggested by McCord and Olson^1^, which describes a transformation process composed of 4 phases (Box). Using this roadmap, health planners may choose the integrated phase as their endpoint. Unfortunately, doing so does not tap the benefits that multiplicity offers in reducing the risk of dependency on a single logistics pipeline and the benefits that competition offers in reducing the number of societal resources spent on ensuring health commodity security.^2^

BOX Supply Chain Evolutionary Roadmap for Health Planners in Africa

1. The **ad hoc phase** is characterized by unstructured, ill-defined, or absent formal logistics functions with fragmented efforts to execute the functions of product selection, quantification, procurement, inventory management, and service delivery. In this phase, performance measurement is not the norm and logistics functions are executed without standard guidelines or operating procedures.
2. The **organized phase** has formal, well-defined logistics functions and a logistic system mapped out. There is sufficient mobilization of resources (capital) to support standardized procedures for executing all logistics functions. These procedures are followed routinely by logistics personnel.
3. The **integrated phase** has separate institutions performing various logistics functions that are coordinated and managed as 1 system. These institutions are linked to other relevant actors: drug regulatory authorities and their quality assurance laboratories, health insurance financing systems, and health care providers. Outsourcing logistics activities (storage, transport, or procurement) to private third-party logistics providers may be considered where appropriate.
4. The **extended phase** has competition-driven alignment of incentives and demand visibility across supply-chain entities, including multiple distributors, suppliers, and manufacturers. In this phase, there are feasible partnerships and collaboration between public, private, or nongovernmental logistics institutions or systems.^1^^,^^2^

Source: McCord and Olson.^1^

However, in moving from the ad hoc to the extended phase of the roadmap, one may ask: What institutional arrangements should be present at that stage of the roadmap, and how can they be developed? A detailed answer to this question should consider all the constituent functions of logistics systems and supply chains. However, for brevity and clarity, this article will focus on the logistics function of inventory management (warehousing, storage, and distribution) that follows product selection, demand quantification, and procurement but precedes service delivery. For the scenarios in this article, I assume that there have been no failures in product selection, demand quantification, and procurement; the needed health commodities have been procured; and the only concern is inventory management failures nullifying all other attempts made to ensure reliable supplies. Given this article’s focus on inventory management, a move toward the extended phase of McCord and Olson’s^1^ roadmap will require health planners to first find arrangements that best serve public interests of ensuring health commodity security. Having identified such arrangements, health planners can initiate reforms to transform the status quo in their respective countries into the desired arrangements.

This article supports a state of prudent multiplicity in that it offers choice, minimizes supply disruptions, and encourages competition. A detailed description of prudent multiplicity is found elsewhere.^3^^,^^4^ In brief, prudent multiplicity is a state in which there are at least 2 competing full-line national logistics systems serving government, nongovernmental, and private health facilities. “Full-line” means each of these competitors is capable of supplying all essential health commodities (whether they require cold-chain storage or not). “National” indicates a capability to serve all health facilities in different geographical locations. Health planners determine the ideal number of competitors when the incremental benefits of having an additional full-line national logistics provider do not outweigh the additional costs.

In the subsequent sections, I explore how health planners can move toward a state of prudent multiplicity. I describe past efforts to improve public-sector supply logistics and how African countries can transform existing public-sector supply logistics and private wholesaling markets to ensure uninterrupted supplies of all health commodities year-round for everyone.

## TRANSFORMING PUBLIC-SECTOR LOGISTICS

### Reconfiguring Public-Sector Monopoly

Public-sector logistics systems in African countries can be described as monopolies because they handle a predominant share of health commodity supplies without the risk of losing business to competitors providing similar services.^3^ Contrary to what one would expect of a monopoly, public-sector supply logistics also lack adequate capital and human investments to provide uninterrupted supplies of health commodities.^5^ Nevertheless, most attempts at improving how public-sector logistics monopolies work have centered on the inefficiencies of having multitiered logistics systems and the underinvestment problems created by spreading limited public financing over multiple tiers or echelons.

The Table shows different multitiered hub-and-spoke arrangements in select African countries based on published data.^5^^–^^12^ The 1-tier and 2-tier arrangements can be considered variations of the 3-tier design in which health commodities are stored and distributed from central medical stores (CMSs) to regional medical stores and then to district medical stores before delivery to health facilities.^5^ These designs are different solutions that countries have pursued or adopted to improve the performance of public-sector supply logistics. Therefore, these designs should not be seen as a prescription for all African countries; if anything, they can be improved. As Clarke and Scarf argued,^13^ the optimal solution (defined in terms of order quantities, output levels, and total costs) for a logistics system with a certain number of tiers or echelons can be replicated by an alternative system with a lower number of tiers or echelons, what is generally referred to as the “system design approach.”^14^ As Fritz et al. reported,^15^ even a simple system-design change, such as switching from manual to electronic logistics management information systems by reducing the frequency of stock-outs of lifesaving health commodities, lowered the mortality rate of newborns and children aged younger than 5 years in Ethiopia, Mozambique, and Tanzania (a reduction of 1.6% to 4.1%). As much as system-design change is desirable, health planners may have to settle for second-best options (and accept efficiency losses) where change is not feasible or there are limits on what can be implemented.

For example, in attempts to redesign a 3-tier public-sector logistics system that is structured to match or reflect the tiers or echelons of governance or public administration, an efficiency loss will have to be accepted if ignoring or working around administrative boundaries is not politically feasible. This is most likely in decentralized or conflict-prone environments. Efficiency losses can appear even in situations in which administrative boundaries can be ignored. The problem is that health planners typically do not have ex ante knowledge about the long-run average cost curves for their logistics systems, nor do they know whether these long-run cost curves are continuous or discontinuous. When such prior knowledge exists, it can be used in planning. However, when that knowledge is not available, it is advisable for planners to conduct baseline costing exercises to gain a deeper understanding of logistics costs and the key drivers. These exercises should be followed by continuous experimentation (trial and error) to determine the optimal scale and scope of operations. Experimentation could start by evaluating several discrete design sizes, such as direct delivery from the CMS to health facilities or a 1-stop (2-tier) delivery strategy. This recommendation fits with the results of a randomized trial conducted in Zambia^16^ that showed direct delivery and a 1-stop (2-tier) delivery strategy that involved a commodity planner who coordinated supply logistics at the district level performed better in reducing the frequency and duration of stock-outs than a 2-tier design that did not employ a commodity planner. In this study, direct delivery to health facilities from Zambia’s Medical Stores Limited—a parastatal or quasi-government CMS—used the district medical stores as a transit (cross-docking) point but not for storage, picking, and packing.^16^

Conducting randomized trials in each low- and middle-income country to identify the optimal system design is a rather costly venture. In fact, some might argue that randomized trials are not required because there are alternative ways of experimenting and searching for the optimal system design. The first approach applies a multicriteria decision-making process (analytic hierarchy process) to rank alternative logistics arrangements based on the priorities and preferences of relevant stakeholders. This approach has been used in Morocco.^11^ A second less sophisticated approach constructs composite indices of volumes of throughput, size of populations served, storage capacity, and distance to service delivery points to determine tiers, distribution centers, or warehouses that need to be eliminated.^17^ A third approach uses mathematical programming or simulation modeling to assess differences in outputs and costs associated with different system designs. This requires prior estimates of logistics costs and an understanding of cost drivers. Activity-based costing, which involves measuring the costs of all activities and steps that comprise a task, will be particularly useful in generating credible parameter values for mathematical programs and simulation models. An example is the simulation model by Lee et al.^18^ Using a sample of 35 countries with 3-tier arrangements (or 4-tiers if including health facilities), the study showed that a simplified 2-tier arrangement with 7 storage hubs or depots could lower operating costs to the extent that, even if larger capital expenditures are required, this design change could still have lower logistics costs per dose of vaccine delivered. Note that this result does not include savings in capital expenditures achievable through redeployment of nonspecific (storage, warehousing, and transportation) assets during the system-design change.

Increasingly, results of mathematical programming or simulation modeling of a finite number of scenarios are used to inform pilot projects before nationwide scale-up, as in Benin, Mozambique, and Niger. Application of these techniques helps reduce the amount of resources expended on experimentation. The scenarios analyzed do not have to be restricted to considering a reduction in the number of tiers; other configuration changes, such as delivery frequency, transportation modes, and routes, can be considered simultaneously.^19^^–^^21^

Next, I argue that although recent experimentation and pilot projects to improve public-sector CMS logistics are a step in the right direction, these efforts are not enough. System-design change should be defined by a continuous search for the optimal scale and scope of operations that ensures 100% availability of essential health commodities in all health facilities most of the time. Do public-sector CMS monopolies have adequate incentives to continuously search for ways of reducing costs and improving outputs?

### Introducing Competition and Self-Financing

As with private wholesalers, well-functioning and efficient public-sector logistics systems have to run an appropriate scale of operations. Over time, they have to secure a sequence of short-run prices for inventory management (i.e., distribution markups) that allows full recovery of operating costs plus the costs of all continuing and repeated investments made to ensure commodity security. If the costs cannot be recovered in the long run through a series of short-run prices, public-sector logistics institutions will have to find alternative financing (e.g., taxes and loans) to cover all the costs of ensuring commodity security. The alternative is injecting public financing to cover costs, but this weakens incentives to pursue efficiency, as public-sector institutions simply spend the money they receive. Although activity-based costing can be used to estimate inventory management costs and identify pockets of inefficiencies within the logistics system, public-sector logistics systems rarely conduct or are rarely required to carry out such costing exercises. In contrast, the quest for survival and/or the competitive pursuit of profits by private wholesalers generates strong incentives to conduct such costing exercises, lower costs, and improve outputs.^4^ Therefore, public-sector logistics systems will require more than just extra funding and resources to improve outputs to desired levels (e.g., ensuring near 100% order fill rates, shorter supply lead times, less frequent stock-outs, shorter spells of stock-outs, and near zero stock expired or damaged).

Public-sector logistics systems (which are more or less public wholesalers) should be allowed to compete with alternative producers of the bundle of services they provide if health planners want to generate strong incentives for the continuous search for new ways of reducing costs and improving service levels (with or without the aid of mathematical programs and simulation models). The “new ways” could be identifying the optimal location, size, and number of warehouses; providing extensive staff training and supervision; using electronic information management systems for more routine data collection; using logistics data for decision-making and evaluation; and automating and experimenting with alternative inventory control policies, transportation modes, distribution routes, and delivery schedules. However, the mere presence of competing alternatives will not put adequate competitive pressure on a public CMS monopoly. In the absence of repeated requests or calls for last-mile delivery and shorter delivery times or generally, if health care purchasers and providers are not sensitive to prices and/or outputs, there will be little or no reason to lower costs and/or upgrade logistics infrastructure for higher service levels even if the money and resources needed were available. Given the emergence of competitors, health care purchasers and providers will have to be sensitive to both price and output to break the dominance of a CMS monopoly.^4^

Public-sector logistics systems should be allowed to compete with alternative producers if health planners want to generate strong incentives for the continuous search for new ways of reducing costs and improving service levels.

Subjecting public-sector logistics systems to competition is not the only way to improve logistics performance. Results-based financing (RBF) of public-sector logistics systems can improve performance if these institutions have full discretion as to how performance-tied incentive payments are used. The additional funding, shared with logistics staff and invested in logistics infrastructure, provides strong incentives to improve specific, verifiable, achievable, and time-bound performance indicators. As Spisak et al. reported,^22^ a 1-year pilot RBF of the CMS in Mozambique improved the number of reports submitted on time and the percentage of inventory records that match physical inventory counts, reduced the time from receipt of orders to completion of distribution planning and from completion of distribution planning to delivery of commodities, and improved the percentage of health commodities delivered that match the packing list in distribution plans. Thus, through RBF of the CMS monopoly, a lot can be achieved without alternative competitors. So, why opt for competition?

In contrast to RBF or performance-based contracting with a single CMS entity, competition requires health planners to enter into a number of performance-based contracts with a multiplicity of logistics providers. To encourage continued efficiency gains, contracted logistics providers who cannot ensure health commodity security and/or charge lower prices for inventory management should lose business to those who can. The difference is the risk-reduction benefits of having more than 1 logistics unit. When a fire crippled Ghana’s CMS, supply disruptions were mitigated by international (nongovernmental) agencies building a parallel logistics system that used a private-sector logistics provider to maintain continuity in health commodities supply.^12^ Having 2 or more logistics institutions capable of full-line stocking and last-mile delivery is an insurance policy against unexpected catastrophic supply disruptions. In contrast, the CMS monopoly model offers no scope for hedging supply disruption risks but ample room for deviations from best standard practices in frantic attempts to reverse the negative impacts of disruptions when they occur.^4^

To promote choice and competition, health planners may add another public-sector logistics system to the existing CMS monopoly. In the Democratic Republic of the Congo and Nigeria, where there are 2 or more CMSs (Table), it might be possible to carve out at least 2 competing public-sector logistics systems from the existing infrastructure. However, given current underfunding problems, creating 2 or more public-sector logistics systems is questionable, especially when similar logistics systems exist or can be built in private markets. An alternative solution is introducing competition between segments of public-sector logistics systems. For example, Watson and McCord^23^ discussed alternative arrangements to a CMS monopoly that included having a competing or complementary parallel CMS. Putting aside concerns about redundant capacity and nonoperational supply lines, that arrangement only offers scope for competition between the central warehouses with little or no effect on supply disruptions that occur at tiers below central warehouses. Such downstream disruptions can be minimized by reducing or removing the tiers below the CMS level, but this will not be enough if disruptions affect all central warehouses or medical stores. Instead of increasing the number of subunits within (tiers of) an existing CMS monopoly, a separate competing logistic pipeline is needed.

**TABLE.** Configuration of Multitiered Public-Sector Health Supply Logistics Systems in Selected African Countries^a,b^

Design	Country	Central Medical Stores	Regional Medical Stores	District Medical Stores
3-tier	Benin^c^	1	8	80
	Burkina Faso	1	7	63
	Democratic Republic of the Congo	2	15	228
	Guinea	1	8	38
	Madagascar	1	22	114
	Mali	1	5	49
	Mozambique^d^	2	11	148
	Niger	1	8	72
	Nigeria^e^	7	36	774
	Senegal	1	11	63
2-tier	Burundi	1	17	–
	Cameroon	1	10	–
	Chad	1	22	–
	Ghana	1	5	–
	Tanzania	1	8	–
	Republic of the Congo	1	–	41
	Rwanda	1	–	30
	Uganda	1	–	112
	Zambia	1	–	72
1-tier	Kenya	1	–	–
	Morocco^f^	1	–	–

a Data from: Yadav, et al.,^5^ Prosser et al.,^6^ Prosser et al.,^7^ Sarley et al.,^8^ Wright et al.,^9^ Yadav,^10^ El Mokrini et al.,^11^ Gavi, the Vaccine Alliance.^12^

b For each country, the number of central medical stores (CMSs), regional medical stores (RMSs), and district medical stores (DMSs) does not include health facilities as a tier or secondary (product-specific) warehouses set up for vertical health programs. The current situation in the countries listed may be different and sometimes, conflicting evidence is reported in the literature.

c In Benin, the number of RMSs refers to 7 department stores and 1 “regional” store; the number of DMSs refers to subdistrict (commune) stores.

d The description of the situation in Mozambique by Wright et al.^9^ differs from Prosser et al.,^7^ who report 1 national warehouse, 9 provincial medical stores, and 128 district stores.

e The CMS tier in Nigeria has 1 national store in Abuja and 6 zonal stores, 1 in each of its 6 geopolitical zones.

f From El Mokrini et al.,^11^ it is unclear whether Morocco has 1 CMS plus 3 secondary warehouses or 2 CMSs plus 2 secondary warehouses.

There is a pro-competition solution that maintains adequate protection against supply disruptions. The first step is transforming a public-sector CMS monopoly into a full-line national entity capable of supplying all health commodities needed to all health facilities in private, government, and nongovernmental sectors. Indeed, the current wave of integrating public-sector logistics pipelines for vaccines and non-vaccines and pharmaceuticals and non-pharmaceuticals in African states and other low- and middle-income countries^24^ is a trend toward full-line supply. Where such public-sector CMS offering integrated, full-line logistics exist, they should be reconfigured to provide national coverage by serving all government, private, and nongovernmental health facilities located in different geographical areas. This requirement should not be viewed as attempting to force 3 sectors into a single system. Despite their commonalities, objective functions, institutional structures (for procurement), and incentives differ across these sectors. The requirement only ensures a set of competing logistics providers have access to the largest possible country demands for health commodities by serving public, private, and nongovernmental sectors. As one of these logistics providers, a transformed public-sector CMS should be able to secure a sequence of prices for the services offered, such that in the long run, they can cover their operating costs and capital investments made without public financing. Self-financing full-line national supply logistics systems may not be feasible in all country contexts. But where it is feasible, health planners should consider turning the transformed public-sector CMS into a fully private or quasi-government unit by ceding management to private agents or through establishing public-private ownership. The choice between transformations of a CMS monopoly into a fully private or quasi-governmental entity will depend on technical, administrative, and political feasibility. For example, having a quasi-government status will be a more workable solution in the face of opposition from proponents of government ownership of supply logistics.

An example of the solution proposed is Zambia’s Medical Stores Limited, which has a mandate to provide last-mile delivery and move to a “fully commercial, self-financing model.”^12^ More recently, in June 2020, Benin’s Central Purchasing Office for Essential Medicines and Medical Consumables underwent a transformation into a public limited company, the Societé Béninoise pour l’Approvisionnement en Produits de Santé (SoBAPS, Benin Society for Health Product Supply).^25^ Currently, there is no evidence on the performance of the SoBAPS, and Zambia’s Medical Stores Limited has been the subject of a number of system-design changes.^16^ Extending these examples to other African countries will require cautious implementation through pilots and careful modifications to reflect differences in local contexts.

An equally important point is that simply privatizing a public-sector CMS monopoly is not the answer. Transforming a public-sector CMS monopoly into a full-line national private (quasi-government) entity should be integrated with attempts to turn existing logistics institutions in private markets and nongovernmental voluntary sectors into credible competitors. Because the competing private or quasi-government logistics units are meant to be self-financing, health planners need to create a level playing field for all competitors, whether private or quasi-government. For example, a quasi-government wholesaler should not benefit from any preferential tax rules unless similar provisions are made for their private competitors.

An alternative strategy is to skip the step of transforming a public-sector CMS and instead focus solely on developing a set of competing full-line national logistics providers in private markets. Existing public-sector logistics infrastructure will then have to be decommissioned and nonspecific assets deployed elsewhere. Because decommissioning a public-sector CMS is unlikely to receive strong political support, the views expressed in this article favor transforming a public-sector CMS monopoly into a full-line national logistics provider and building competing alternatives to the transformed CMS from private markets.

## USING PRIVATE WHOLESALERS

### Private Markets in Africa Failing to Ensure Commodity Security

**FIGURE 1:**
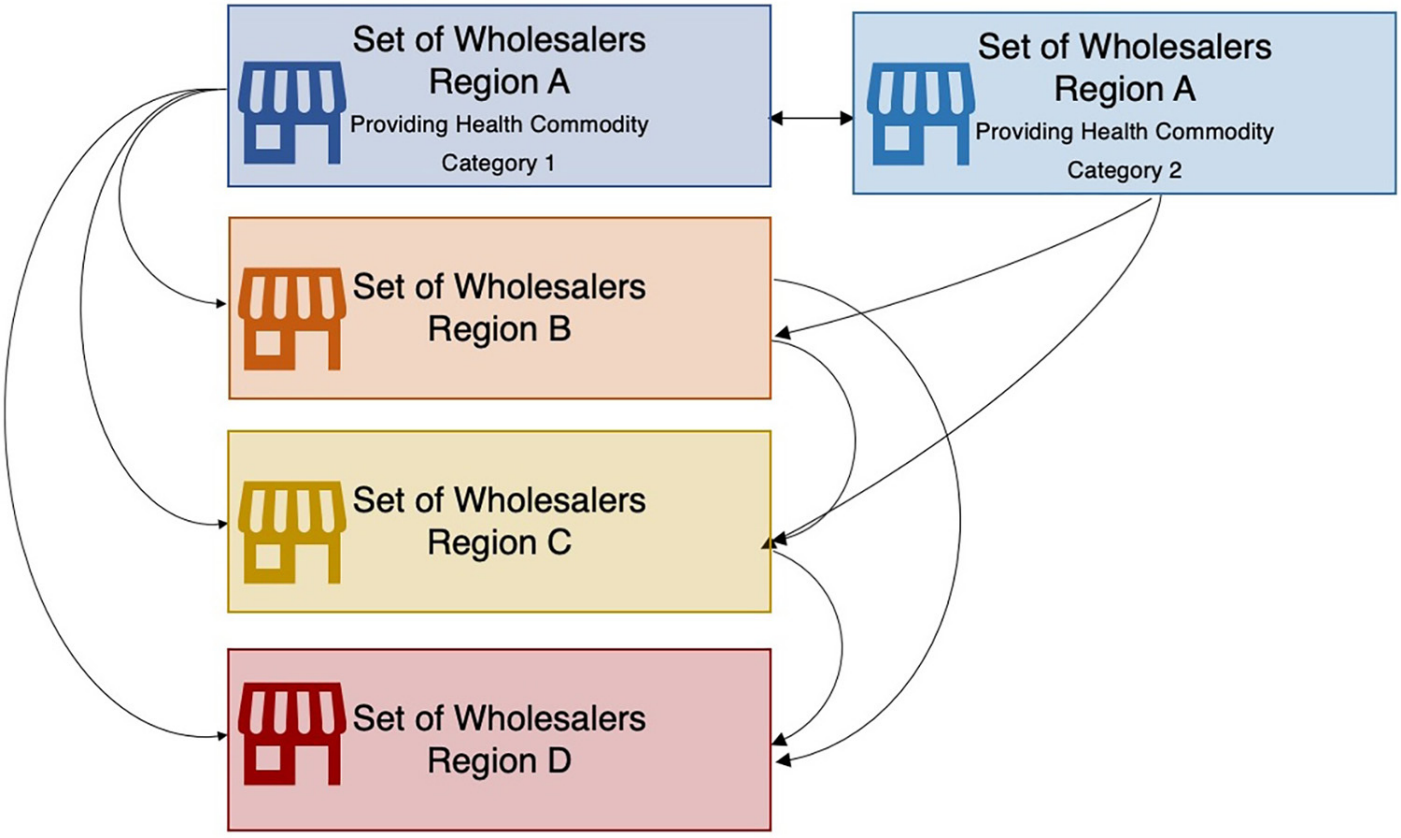
Transactions of Health Commodities Between Short-Line Subnational Private Wholesalers Operating Within Regions of a Given Low- and Middle-Income Country in Africa^a^ Source: Author; Yadav et al.,^5^ Palafox et al.,^26^ Rosen et al.,^27^ McCabe et al.^27^

In Africa, underfunded public-sector logistics systems sit next to small-scale, short-line, subnational private wholesalers who do not serve rural or remote areas and survive on high margins on commodity acquisition prices. According to the World Health Organization,^5^ Uganda has 100 officially registered wholesalers, with 12–14 being the “big players”; Nigeria has 292 importer-wholesalers; and Zambia has 30 licensed wholesalers, with business volumes concentrated among 5 or 6 wholesalers. In Francophone Africa, the average is 5 wholesalers/importers per country, with the exception of Burundi (14) and Rwanda (32). In Kyrgyzstan, there are 200 private wholesalers, with only 10 considered “volume leaders.” In contrast, the average across Organisation for Economic Co-operation and Development countries is 3–5 large private wholesalers who can deliver medicines several times a day to ensure health commodity security.^5^ According to Palafox et al.,^26^ the multiplicity of formal and informal private antimalarial wholesalers in Benin, Cambodia, Democratic Republic of the Congo, Nigeria, Uganda, and Zambia is such that health commodities go through 4–6 steps (2–3 steps in most cases) in moving from manufacturer to retailers. Intermediate private wholesalers selling health commodities to each other before selling to end users is evidence that they are mostly short-line and/or subnational entities. Although this can be described as multiplicity, it does not encourage competition in ensuring health commodity security or minimizing supply disruption risk (Figure 1).^4^

The system of numerous middlemen or brokers undermines health commodity security in 2 ways. First, serial markups levied on manufacturer selling prices make end-user prices unaffordable. Private wholesaler markups in African countries range from 2% to 380% such that manufacturer selling prices could represent as little as 8% of final patient price.^27^ Second is unreliable service levels. For example, private wholesalers in urban areas in Ghana receive orders from health facilities, but those facilities in periurban and rural areas (in the Northern regions) have to travel to commercial areas or hubs in the capital and other major cities.^28^ As Palafox et al. reported,^26^ the practice of “way billing” (i.e., billing for transportation) in which health commodity orders/supplies are placed on bus or mass transport routes for customer pickup at regional bus or taxi stations is quite common in African countries. The small scale of operations of private wholesalers in Africa is such that they do not usually offer last-mile delivery services, so their customers have to pick up the commodities themselves. The adage “if the drugs [health commodities] won’t come to you, you go for the drugs [health commodities]” is a sign and symptom of gaps in commodity security. These gaps often invite individual salesmen to fill their vans with health commodities, traveling countrywide to sell these commodities to rural pharmacies and chemical sellers.^28^

Some might argue that numerous differentiated short-line private wholesalers are more suited for the supply of different health commodities with different storage requirements (some need cold-chain equipment and others do not). But given a choice between full-line national wholesaling (a set of long-distance distributors running up and down the country) and short-line subnational wholesaling (a set of short-distance distributors operating locally or regionally), the latter arrangement is more likely to result in gaps in commodity security. Indeed, 1 private-market response to these gaps is the emergence of “one-stop-shop” middlemen who recognized that the numerous short-line importers and wholesalers do not offer the wide range of health commodities needed to meet all demands in different geographical locations.^28^

### Steering Private Markets Toward Public Interests

There is some evidence that private wholesaling in Africa can move from what is shown in Figure 1 toward a state of prudent multiplicity without government intervention. In an attempt to broaden its product portfolio and scale of operations, a company called Gokals (Laborex Ghana) merged with or acquired several small-scale private wholesalers, demonstrating that it is possible to consolidate small-scale private wholesalers across different regions of the country into national full-line entities or at least logistics institutions with a broader portfolio of health commodities and a wider geographical coverage. That said, Gokals holds exclusive distribution rights over selected health commodities sourced through Laborex, raising some anticompetitive concerns. These exclusionary arrangements may be a response to low expected business volumes and/or attempts to cover the additional (quasi-fixed) costs of getting health commodities in-country within 24 hours. Supply lead times of small-scale private wholesalers are a lot longer as they rely mostly on sea routes in sourcing their products.^28^ However, the emergence of similar large-scale private wholesalers in Ghana will require revoking any existing exclusive (geographic) distribution rights for health commodities because it works against competition and acts as a deterrent to market entry and growth of other private wholesalers aiming to transform their businesses into full-line national logistics units.

**FIGURE 2:**
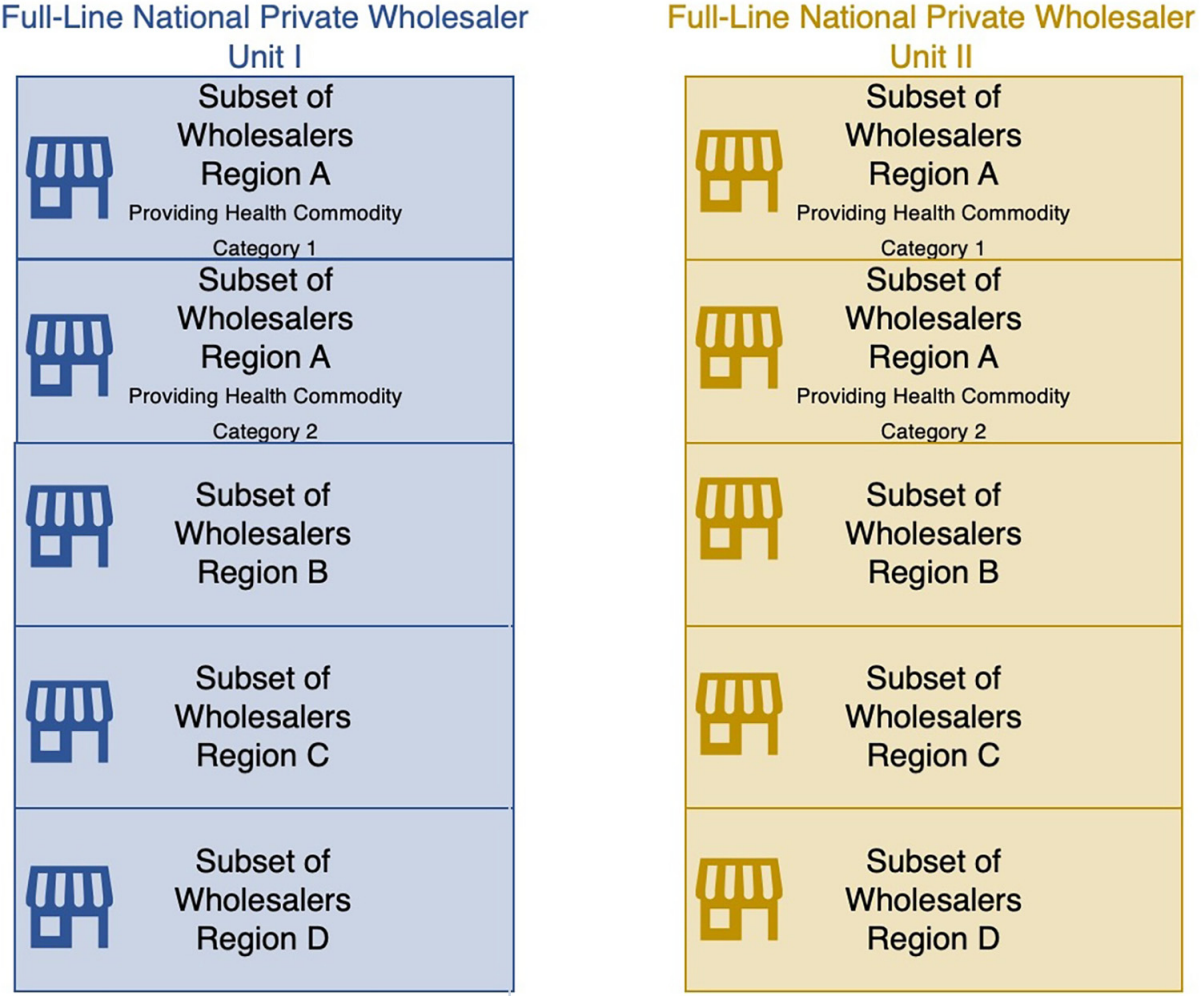
Transformation of the Multiplicity of Short-Line Subnational Private Wholesalers to Form Multiple Competing Full-Line National Logistics Units in a Low- and Middle-Income Country in Africa

Generally speaking, government intervention (legislation, policy, and regulations) will be needed to initiate the process of transforming a public-sector CMS monopoly and steering private wholesaling toward the societal goal of ensuring health commodity security. Governments should at least accelerate efforts made by private wholesalers on their own toward full-line stocking and national coverage. For example, the emergence of a full-line national self-financing quasi-government wholesaler may create incentives for private wholesalers to transform themselves into full-line national units. That process could be hastened by removing legislation that requires exclusive use of a public-sector CMS for inventory management and government regulations that require (new) logistics providers to be full-line national carriers, making it impossible for any private competitor to focus on a smaller range of health commodities, serve only selected geographical regions, or serve a subset of health facilities. Figure 2 shows how competing full-line national logistics institutions can be formed out of the short-line subnational private wholesaling arrangements depicted in Figure 1. In Figure 2, short-line subnational private wholesalers collaborate or cooperate with each other to form a minimum of 2 or more full-line national logistics providers (Unit I and Unit II). The separate full-line national logistics units created compete to deliver all essential health commodities demanded nationwide. Short-line subnational entities that are members or affiliates of the same full-line national unit do not compete with each other. These private entities could be either pure wholesalers (who only manage inventory) or wholesalers who are also importers and procurement agents. The key requirement is that their combined capacities should allow the delivery of a full range of inventory to all health facilities in different geographical regions.

Government intervention will be needed to initiate the process of transforming a public-sector CMS monopoly and steering private wholesaling toward the societal goal of ensuring health commodity security.

For example, in Benin, there are 5 formal private wholesalers (4 before 2014) that are by law required to import and distribute the same pharmaceutical products: at least nine-tenths of all registered products in the country.^25^^,^^29^ However, these wholesalers are only allowed to supply pharmacies, public health centers, and “other pharmaceutical establishments” but not private clinics or warehouses (depots) in rural areas, a restriction that appears to have created a sizable and thriving informal drug market. In fact, adding informal wholesalers supplying registered and unregistered antimalarials to the 5 formal private wholesalers increased the number of “wholesalers” in Benin to more than 200 without a corresponding improvement in health commodity security.^26^ I will argue that the requirement in Benin for wholesalers to supply at least 90% of all registered medicines should be maintained, as it ensures any new entrant into the formal Beninese wholesaling market will at least be a full-line logistics institution. In addition to this measure, regulations should be relaxed to allow formal private wholesalers to serve all governmental, private, and nongovernmental health facilities in different geographical locations. After adding SoBAPS (the newly created public limited company with the Beninese government as the sole shareholder), it is possible to have 6 full-line national logistics institutions competing to ensure uninterrupted supplies of health commodities in Benin.

It is worth mentioning that in Benin, as in most Francophone Africa, there is no wholesaler markup competition. Private wholesaler markups are fixed by regulation,^27^ which might hinder attempts to build competing full-line national private wholesalers in Benin. This is because the free setting of wholesaler markups and consumer prices, as well as the removal of any monopoly licenses or exclusive distribution rights over specific products, expands the ability that private wholesalers have to recover any continuing or repeated capital investments that are made to ensure commodity security (e.g., providing just-in-time deliveries even in rural areas). Private wholesalers will be more willing to make such investments if the associated costs can be recouped through flexible prices charged for their services. Competition driven by health care payer and provider price sensitivity should then keep prices for inventory management low. End-user prices can equally be kept low if there is also competition between manufacturers to reduce procurement costs and competition to lower retail pharmacy margins.

## CONSIDERING POTENTIAL BARRIERS TO A STATE OF PRUDENT MULTIPLICITY

First, I described an evolutionary path or roadmap^1^—which is different from X-year roadmaps (or master plans) developed by domestic and external health planners for specific regions, countries, or a single country—that was chosen because it is not context specific. This roadmap shows (from the ad hoc to the extended phases) a sequence of investments to reach a state in which there is a multiplicity of and competition between supply chains to support health programs’ objectives of delivering value at a level that is greater than the sum of parts.

Focusing on the logistics function of inventory management, I described how a state of prudent multiplicity can be implemented. Health planners should consider (in their roadmaps, master plans, or health system strengthening initiatives) creating 2 or more self-financing full-line national logistics institutions that compete against each other. This section explores 4 barriers health planners should be aware of before adopting a state of prudent multiplicity.

First, this proposal should not be considered in isolation. What the logistics system actually looks like and how it operates will not be determined by distribution (inventory management) policy alone. Indeed, the impetus to reform logistics systems at the country level will be absent without public outcry for change and a supportive political climate for broader health system reforms. Here, I restricted my focus to the inadequacies of current distribution arrangements in which public-sector logistics providers serve mostly public, faith-based, and nongovernmental health facilities, and private wholesalers serve mostly private health facilities or concentrate their businesses on niche commodities with lower demand volumes managed outside of national competitive tenders. I proposed transforming these fragmented logistics pipelines into a set of full-line national logistics providers that serve all public, private, and nongovernmental health facilities. This solution should be tailored and fitted to policy reforms related to product selection, demand quantification, procurement, and service delivery. Similarly, health planners must find ways of fitting this proposal into existing decentralized arrangements. For full-line logistics providers to offer national coverage, their operations must transcend decentralized administrative boundaries. As noted earlier, an efficiency loss will have to be accepted if this is not possible. In such situations, health planners may settle for full-line subnational arrangements operating within each decentralized administrative region, but I believe there is greater scope for gains if the competing units can be full-line and national without conflicts with decentralization.

Transforming fragmented logistics pipelines into a set of full-line national logistics providers that serve all public, private, and nongovernmental health facilities should be tailored and fitted to policy reforms related to product selection, demand quantification, procurement, and service delivery.

Second, successful privatization must consider multiple factors. Although Zambia and Benin have transformed their public-sector CMS into private entities, governments in other countries may be uncomfortable moving away from nationally owned logistics institutions. Moreover, evidence from non-African settings on the successful privatization of public-sector supply logistics is unconvincing. For example, in Malaysia, after privatizing the state-run general medical stores in 1994, prices increased 10.42% in 1995–1996, decreased 3.37% in 1997–2000, and increased 64.04% in 2001–2003.^30^ These findings cast doubts on the benefits of privatizing public-sector supply logistics. However, it is important to note that, in Malaysia, privatized general medical stores signed a 15-year contract to execute the logistics functions of procurement and inventory management. Because end-user prices are the sum of procurement prices and prices for inventory management, gains in inventory management can be negated or masked by procurement failures. What is more, turning a public monopoly into a private monopoly (as in Malaysian general medical stores) will not yield the expected outcomes without multiple competing logistics units for inventory management and/or procurement practices that are consistently guided by national or international competition. Choosing the integrated phase of McCord and Olson’s^1^ roadmap as the endpoint ignores opportunities for additional welfare gains from multiplicity. However, some caution needs to be exercised in moving to a state of prudent multiplicity. As recommended by the World Health Organization,^5^ competition and cost containment should not be implemented too early (and perhaps too quickly) because of the large capital outlays and investments in human resources needed to develop 2 or more competing full-line national logistics institutions. Early implementation of cost containment may jeopardize the growth and development of the desired form of multiplicity. Delaying competition will give the logistics institutions that are created more time to fully recoup all fixed, common, or sunk-cost investments made. Determining how long competition should be delayed is an open question but requires working with a longer planning horizon (e.g., a 10- or 20-year roadmap) and ensuring delays do not stifle competition.

Third, private wholesalers may not respond to governments’ efforts to create full-line national logistics institutions. As Yadav et al. noted,^31^ in low- and middle-income countries, it is more difficult for private wholesalers to ensure continuous supplies of essential medicines than soft drinks. Unlike health commodities, soft drinks are consumed whether people fall sick or not. The higher sales revenue lost from stock-outs and competition from substitute kola products provides strong incentives for an uninterrupted supply of soft drinks. Similar incentives for an uninterrupted supply of health commodities are absent given unpredictable demands, positive herd-immunity effects that reduce future demands, and negative effects of excessive usage (e.g., development of treatment resistance) that require constraints on per-person consumption and sales. Furthermore, unlike essential health commodities, the distribution of soft drinks can be easily pooled with other non-health consumer products. Thus, the emergence and survival of full-line national logistics providers is likely to be hampered by cash flow and financing difficulties, especially where delays in payments for logistics services rendered are almost a cultural norm. As a countermeasure to these difficulties and to ensure supply logistics is a profitable venture without irrational consumption of health commodities, it is crucial that the set of competing logistics units supply the full range of health commodities and serve all government, private, and nongovernmental health facilities in a given country. Doing so presents the largest possible country demand and offers opportunities to supplement poor revenues with good revenues on specific commodities to achieve positive overall profits. However, this measure will not work if nonpayment or payment delays force logistics providers to serve only health facilities and regional counties with a solid credit history. More worrying is that incentives for continuous improvement will be dampened if logistics providers are not guaranteed payments for being efficient. As part of our proposal, health planners should implement accounting and financial systems to address nonpayment or delays in payment.

Finally, it could be argued that poor roads, highways, and public transport infrastructure in African countries are more important constraints on health commodity security than the inefficiencies of public monopoly. But as much as good transport networks are sorely needed, the poor existing transport infrastructure somehow does not undermine the continuous supply of soft drinks or alcoholic beverages nor the transportation of farm produce to cities and ports. Considering the lifesaving benefits provided by health commodities (in emergencies), I argue that the constraints imposed by poor transport networks should be reduced or removed by making additional investments in alternative modes of transport (e.g., use of hired cargo planes, helicopters, drones, or ferry boats). This is more so where flooded roads are rendered impassable during rainy seasons. However, the additional investment costs in alternative transport modes should not be recouped by charging higher markups over a short period of time. Markups could be kept low by spreading costs over longer time periods and demands in all 3 sectors. If mobilizing funding for such long-term investments is not feasible, then as De Boeck et al. suggested,^32^ health planners may consider alternative short-term strategies. For example, the problem of impassable road networks could be resolved by holding 100% buffer inventory (i.e., roughly doubling forecasted quantities for the rainy season) and increasing the frequency of stock replenishments before the start of and during the rainy season.

## CONCLUSION

To ensure health commodity security in African countries, societal resources should be channeled toward building a set of at least 2 quasi-government and private logistics institutions that compete in serving all private, government, and nongovernmental health facilities. The set of logistics institutions built will be faced with the largest possible country demand to support continuing or repeated sunk-cost investments in (upgrading) logistics infrastructure to reduce costs and improve service levels. Health planners may consider delaying competition in supply logistics, but competition should not be stifled, barred, or avoided. In line with Bornbusch and Bates,^3^ instituting such a state of prudent multiplicity will require health planners in Africa to view current government, private, and nongovernmental arrangements in their respective countries as part of an “overarching [logistics] system” that can be reconfigured and transformed into a set of competing full-line national logistics institutions. Where private wholesaling markets, on their own, show signs of moving toward a state of prudent multiplicity, health planners only have to accelerate that process. If not, governments have to initiate the process of steering private markets toward public interests.

## Notes

**Peer Reviewed**

**First published online:** February 5, 2024.

## References

[1] McCord J, Olson N. *Supply Chain Evolution: Introduction to a Framework for Supply Chain Strengthening of Developing Country Public Health Programs*. USAID DELIVER Project; 2011. Accessed January 16, 2024. http://iaphl.org/wp-content/uploads/2016/05/Suppy-Chain-Evolution.pdf

[2] Bornbusch A, Dickens T, Hart C, Wright C. *A Business Approach to Transforming Public Health Supply Systems*. Reproductive Health Supplies Coalition; 2014. Accessed January 16, 2024. https://www.rhsupplies.org/uploads/tx_rhscpublications/A_Business_Approach_to_Transforming_Public_Health_Supply_Systems.pdf

[3] Bornbusch A, Bates J. Multiplicity in public health supply systems: a learning agenda. Glob Health Sci Pract. 2013;1(2):154–159. 10.9745/GHSP-D-12-00042. 25276528 PMC4168568

[4] Tetteh EK. Consolidation or multiplicity in supply logistics for health commodities? Explor Res Clin Soc Pharm. 2022;5:100105. 10.1016/j.rcsop.2022.100105. 35478501 PMC9031374

[5] Yadav P, Tata HL, Babaley M. *World Medicines Situation 2011: Storage and Supply Chain Management*. World Health Organization; 2011. Accessed January 16, 2024. https://www.asrames.org/wp-content/uploads/2016/07/THE-WORLD-MEDICINES-SITUATION-2011-STORAGE-AND-SUPPLY-CHAIN-MANAGEMENT.pdf

[6] Prosser W, Jaillard P, Assy E, et al. System redesign of the immunization supply chain: experiences from Benin and Mozambique. Vaccine. 2017;35(17):2162–2166. 10.1016/j.vaccine.2016.09.073. 28364925

[7] Prosser W, Folorunso O, McCord J, et al. Redesigning immunization supply chains: results from three country analyses. Vaccine. 2021;39(16):2246–2254. 10.1016/j.vaccine.2021.03.037. 33752952

[8] Sarley D, Mahmud M, Idris J, et al. Transforming vaccines supply chains in Nigeria. Vaccine. 2017;35(17):2167–2174. 10.1016/j.vaccine.2016.11.068. 28364926

[9] Wright M, Forster G, Beale J. Improving iSC performance through outsourcing – Considerations for using third-party service providers to increase innovation, capacity and efficiency. Vaccine. 2017;35(17):2195–2197. 10.1016/j.vaccine.2016.11.108. 28364930

[10] Yadav P. Health product supply chains in developing countries: diagnosis of the root causes of underperformance and an agenda for reform. Health Syst Reform. 2015;1(2):142–154. 10.4161/23288604.2014.968005. 31546312

[11] El Mokrini A, Benabbou L, Berrado A. Multi-criteria distribution network redesign - case of the public sector pharmaceutical supply chain in Morocco. Supply Chain Forum 2018;19:42–54. 10.1080/16258312.2018.1433436

[12] Gavi, the Vaccine Alliance. *GAVI Study: Outsourcing the Distribution Component of Vaccine and Medicine Supply Chains*. Gavi; 2015. Accessed January 17, 2024. https://www.transaid.org/wp-content/uploads/2016/02/GAVI-Outsourcing-Report.pdf

[13] Clark AJ, Scarf H. Optimal policies for a multi-echelon inventory problem. Manage Sci. 2004;50(12 Suppl):1782–1790. 10.1287/mnsc.1040.0265

[14] Prosser W, Spisak C, Hatch B, McCord J, Tien M, Roche G. Designing supply chains to meet the growing need of vaccines: evidence from four countries. J Pharm Policy Pract. 2021;14(1):80. 10.1186/s40545-021-00368-x. 34587993 PMC8482642

[15] Fritz J, Herrick T, Gilbert SS. Estimation of health impact from digitalizing last-mile logistics management information systems (LMIS) in Ethiopia, Tanzania, and Mozambique: a Lives Saved Tool (LiST) model analysis. PLoS One. 2021;16(10):e0258354. 10.1371/journal.pone.0258354. 34695158 PMC8544866

[16] Vledder M, Friedman J, Sjöblom M, Brown T, Yadav P. Improving supply chain for essential drugs in low-income countries: results from a large scale randomized experiment in Zambia. Health Syst Reform. 2019;5(2):158–177. 10.1080/23288604.2019.1596050. 31194645

[17] Tetteh E. Creating reliable pharmaceutical distribution networks and supply chains in African countries: implications for access to medicines. Res Social Adm Pharm. 2009;5(3):286–297. 10.1016/j.sapharm.2008.08.001. 19733829

[18] Lee BY, Connor DL, Wateska AR, et al. Landscaping the structures of GAVI country vaccine supply chains and testing the effects of radical redesign. Vaccine. 2015;33(36):4451–4458. 10.1016/j.vaccine.2015.07.033. 26209835

[19] Assi TM, Brown ST, Kone S, et al. Removing the regional level from the Niger vaccine supply chain. Vaccine. 2013;31(26):2828–2834. 10.1016/j.vaccine.2013.04.011. 23602666 PMC3763189

[20] Lee BY, Haidari LA, Prosser W, et al. Re-designing the Mozambique vaccine supply chain to improve access to vaccines. Vaccine. 2016;34(41):4998–5004. 10.1016/j.vaccine.2016.08.036. 27576077 PMC5547748

[21] Brown ST, Schreiber B, Cakouros BE, et al. The benefits of redesigning Benin’s vaccine supply chain. Vaccine. 2014;32(32):4097–4103. 10.1016/j.vaccine.2014.04.090. 24814550

[22] Spisak C, Morgan L, Eichler R, Rosen J, Serumaga B, Wang A. Results-based financing in Mozambique’s central medical store: a review after 1 year. Glob Health Sci Pract. 2016;4(1):165–177. 10.9745/GHSP-D-15-00173. 27016552 PMC4807757

[23] Watson N, McCord J. *Alternative Public Health Supply Chains: Reconsidering the Role of the Central Medical Stores*. USAID DELIVER Project; 2013. Accessed January 17, 2024. https://www.psmtoolbox.org/wp-content/uploads/2017/11/Alte_Publ_Heal_Supp.pdf

[24] Yadav P, Lydon P, Oswald J, Dicko M, Zaffran M. Integration of vaccine supply chains with other health commodity supply chains: a framework for decision making. Vaccine. 2014;32(50):6725–6732. 10.1016/j.vaccine.2014.10.001. 25446826

[25] Mahamé S, Houngnihin RA, Kpatchavi AC. Strengths and weaknesses of State-controlled wholesale distribution: Benin’s CAME and wholesaler-distributors. In: Baxerres C, Cassier M, eds. *Understanding Drugs Markets: An Analysis of Medicines, Regulations and Pharmaceutical Systems in the Global South*. Routledge; 2022:52–93.

[26] Palafox B, Patouillard E, Tougher S, et al. Understanding private sector antimalarial distribution chains: a cross-sectional mixed methods study in six malaria-endemic countries. PLoS One. 2014;9(4):e93763. 10.1371/journal.pone.0093763. 24699934 PMC3974780

[27] Rosen D, Rickford S. *Supply Chain Optimization in Africa's Private Sector: Reducing the Price to Patient*. IMS Health Incorporated; 2014. Accessed January 17, 2024. https://marketbookshelf.com/wp-content/uploads/2017/06/IMS-Health-Supply-Chain-Optimisation-in-Africas-Private-Sector-160514-1.pdf

[28] McCabe A, Seiter A, Diack A, Herbst CH, Dutta S, Saleh K. Private Sector Pharmaceutical Supply and Distribution Channels in Africa: A Focus on Ghana, Malawi and Mali. Health, Nutrition and Population (HNP) Discussion Paper. World Bank; 2011. Accessed January 17, 2024. https://www.jstor.org/stable/resrep26274.1?seq=4

[29] Baxerres C, Le Hesran JY. Where do pharmaceuticals on the market originate? An analysis of the informal drug supply in Cotonou, Benin. Soc Sci Med. 2011;73(8):1249–1256. 10.1016/j.socscimed.2011.03.050. 21962151

[30] Babar ZD, Izham MIM. Effect of privatization of the drug distribution system on drug prices in Malaysia. Public Health. 2009;123(8):523–533. 10.1016/j.puhe.2009.06.011. 19665741

[31] Yadav P, Stapleton O, van Wassenhove L. Learning from Coca-Cola. Stanf Soc Innov Rev 2012;11(1):51–55. 10.48558/2PNM-CA15

[32] De Boeck K, Decouttere C, Jónasson JO, Vandaele N. Vaccine supply chains in resource-limited settings: mitigating the impact of rainy season disruptions. Eur J Oper Res. 2022;301(1):300–317. 10.1016/j.ejor.2021.10.040

